# Risk factors for suicidal behaviours in late-onset bipolar disorder

**DOI:** 10.1192/j.eurpsy.2021.104

**Published:** 2021-08-13

**Authors:** A. Lengvenyte

**Affiliations:** 1 Department Of Emergency Psychiatry And Acute Care, Psnrec, Univ Montpellier, Inserm, CHU Montpellier, Montpellier, France; 2 Faculty Of Medicine, Institute Of Clinical Medicine, Psychiatric Clinic, Vilnius University, Vilnius, Lithuania

**Keywords:** bipolar disorder, Suicide, old age, suicidal behaviour

## Abstract

Late-onset bipolar disorder (BD), when symptoms emerge after the age of 50 years, has gained recognition in the past decades. Currently, BD of about one in ten older patients is considered to be late-onset. Since suicide risk is extremely elevated in BD, especially at the onset of the illness, patients that live to old age are generally considered a survivor population. Meanwhile, patients with late-onset BD did not have BD while living through life periods that could be associated with typical risk factors for suicidal behaviours. Moreover, the late-onset BD might have specific etiopathogenesis, as demonstrated by less genetic component and more life stressors, medical comorbidity and alcohol use. Clinically, patients with late-onset BD have more depressive episodes and more favourable treatment outcomes, yet clinicians generally fail to adhere to guidelines while treating these patients. In n=614 older age BD patients from FondationFondamental Expert Centers, late-onset BD patients reported less lifetime suicidal ideation and attempts compared to non-late-onset patients, while there was no difference regarding the last year suicidal ideation. Better verbal memory was associated with more suicidal behaviour reporting in both groups. Meanwhile, late-onset patients had lower affect intensity and less childhood trauma – factors that were strongly positively associated with last year suicidal ideation in patients with earlier, but not late-onset BD. Meanwhile, late-onset BD patients had higher arterial blood pressure, which was associated with lifetime suicide attempt history in them, but not in earlier-onset patients. Late-onset BD seems to have a distinct pathway to suicidal behaviours.

**Disclosure:**

No significant relationships.

